# Cadaveric Study of the Junction Point Where the Gastrocnemius Aponeurosis Joins the Soleus Aponeurosis

**DOI:** 10.2174/1874325001711010762

**Published:** 2017-07-31

**Authors:** Tun Hing Lui, Chong Yin Mak

**Affiliations:** Department of Orthopaedics and Traumatology, North District Hospital 9 Po Kin Road, Sheung Shui, NT, Hong Kong SAR, China

**Keywords:** Gastrocnemius, Aponeurosis, Recession, Endoscopy

## Abstract

**Purpose:**

To study the location of the junction point where the gastrocnemius aponeurosis joins the soleus aponeurosis to form the Achilles tendon.

**Methods:**

Twelve lower limb specimens were used. The distance between the medial tibial plateau and the superior border of the posterior calcaneal tubercle (A) was measured and the distances of the junction point to the superior border of the posterior calcaneal tubercle (B) were measured.

**Result:**

The ratio B/A averaged 0.45. The gastrocnemius muscle reached or extended beyond the junction point in eight specimens (67%). The average distance from the lowest border of the muscle to the junction point was 0±12mm (-25-25).

**Conclusion:**

There are great anatomical variations of the gastrocnemius insertion. Resection of muscle bound portion of the gastrocnemius aponeurosis is a more appropriate approach of endoscopic gastrocnemius aponeurosis recession.

**Clinical Relevance:**

This report suggests that resection of muscle bound portion rather than the muscle void portion of the gastrocnemius aponeurosis is a more appropriate approach of endoscopic gastrocnemius aponeurosis recession.

## INTRODUCTION

A gastrocnemius equinus is typically characterized by less than 10° of ankle dorsiflexion with the knee extended with positive Silfverskiold test [1]. This can lead to various secondary problems, including Achilles tendinosis, flatfoot, lower back pain or strain, knee hyperextension (genu recurvatum), plantar fasciitis, midfoot pain or arthritis, metatarsalgia, posterior tibial tendon insufficiency, osteoarthritis, and foot ulcers [2-5]. Gastrocnemius recession surgery is performed to weaken or eliminate the gastrocnemius muscle’s plantarflexory action on the foot [6, 7]. Open gastrocnemius recession can be at either the gastrocnemius aponeurosis distal to the gastrocnemius muscle attachment [8-11] or at the anterior surface of the muscle bound portion of the gastrocnemius aponeurosis [1, 6, 12-15]. Endoscopic gastrocnemius recessions have been developed recently and reported to have fewer complications and better cosmetic outcome [2, 3, 16-26]. They have been used as an adjunctive treatment of posterior tibial tendon dysfunction, forefoot nerve entrapment, metatarsalgia, refractory Achilles tendinopathy, cerebral palsy and pediatric pes planovalgus [16, 18-20, 27-29]. The endoscopic techniques are mostly an endoscopic approach of the Strayer-type of complete recession of the gastrocnemius aponeurosis. As a minimally invasive approach, the portal wounds are small and therefore it should be accurately placed at the level of the muscle void portion of the gastrocnemius aponeurosis. We believe that the junction point where the gastrocnemius aponeurosis joins the soleus aponeurosis to form the Achilles tendon is an important landmark. The endoscopic gastrocnemius aponeurosis recession can be performed just proximal to this point. In this study, the relationships between the junction point and the surrounding surface anatomic landmark was studied. We hypothesized that the junction point can be accurately determined by studying the relationship of the point with the other surface landmarks.

## METHODS

**Fig. (1):**
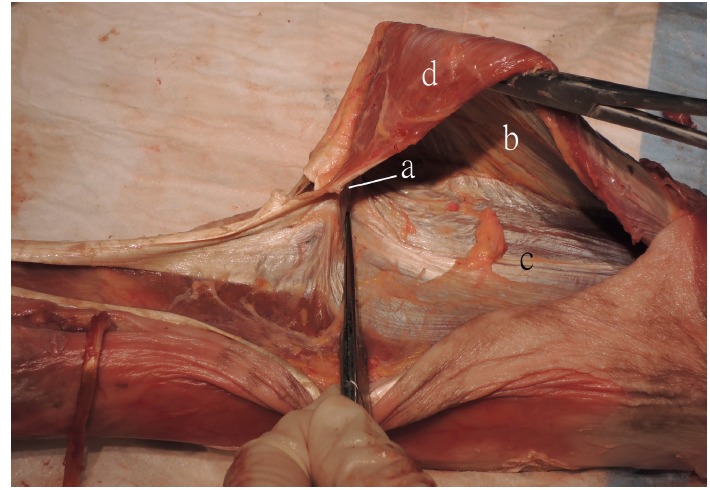
Specimen showing that the junction point **(a)** where the gastrocnemius aponeurosis **(b)** joined the soleus apoeneurosis **(c)**. **d:** gastrocnemius muscle.

Twelve lower limb specimens from 6 fresh frozen Chinese cadavers (5 male and 1 female) were used. The average age of succumb was 79.5 year old (65-91). None of the cadavers had deformity, trauma or any surgery of their lower limb. The skin was resected to expose the muscles, bones and joints. The junction point where the gastrocnemius aponeurosis joined the soleus aponeurosis was identified (Fig. **1**). The distance between the medial tibial plateau and the superior border of the posterior calcaneal tubercle was measured with the ankle and subtalar joints in neutral position. The junction point of the gastrocnemius and soleus aponeuroses was identified and the distance of this point to the superior border of the posterior calcaneal tubercle was measured. The distance from the lowest border of the gastrocnemius muscle to the junction point was also measured.

## RESULTS

The results of the cadaveric study were summarized in (Table **1**).

The average distance between the medial tibial plateau and the superior border of the posterior calcaneal tubercle (A) was 373±27mm (335-415). The average distance between the junction point and the superior border of the posterior calcaneal tubercle (B) was 168±20mm (120-190). The ratio B/A averaged 0.45±0.07 (0.31-0.54). The gastrocnemius muscle reached or extended beyond the junction point in eight specimens (67%). The average distance from the lowest border of the muscle to the junction point was 0±12mm (-25-25).

## DISCUSSION

In this study, the junction point cannot be accurately determined by surface landmarks. Moreover, muscle-void portion of the gastrocnemius aponeurosis that allowing Strayer-type of endoscopic gastrocnemius aponeurosis recession was identified in only four (33%) specimens. In these 4 specimens, the muscle void portion of gastrocnemius sponeurosis was at most 25mm in length, which was a narrow zone that may be difficult to locate accurately during endoscopic gastrocnemius aponeurosis recession.

Endoscopic gastrocnemius aponeurosis recessions allow release of the gastrocnemius aponeurosis under arthroscopic visualization through small portal wounds. The reported techniques target the exposed inferior portion of the aponeurosis that is not directly covered by muscle [2, 3, 16-26]. Different surface landmarks including the distal border of the gastrocnemius muscle [22, 23], the fibula length [30] and the medial malleolus [22] have been used to locate the muscle void portion of the gastrocnemius aponeurosis. However, these localization methods are of doubtful accuracy because of the anatomical variations of the gastrocnemius insertion. The location where the gastrocnemius aponeurosis joins the soleus aponeurosis and the length of the muscle void portion of the gastrocnemius aponeurosis can be variable and the gastrocnemius muscle can even insert directly onto the tendinous superficial surface of the soleus [12]. If the portal wounds are not placed at the level of the muscle void portion of gastrocnemius aponeurosis, the wounds needed to be extended and the goal of “minimally incision surgery” is defeated. On the other hand, endoscopic resection of the gastrocnemius aponeurosis that is covered by the gastrocnemius muscle has a more constant surface landmark [31, 32]. From this study, the muscle-bound portion of the gastrocnemius aponeurosis can be confidently reached if the portal wounds are more than 25mm above the inferior border of the gastrocnemius muscle and will not be affected by the gastrocnemius variable insertion. This approach preserves the insertion of gastrocnemius, allowing for both an intramuscular and aponeurotic lengthening [6, 13]. It can lessen the force applied by the gastrocnemius muscle on the foot without entirely decommissioning the muscle’s biomechanical influence [6]. In contrast to the Strayer-type of endoscopic gastrocnemius aponeurosis recession, preservation of the gastrocnemius insertion allows the gastrocnemius to maintain a “weakened” effect on the foot and the amount of calf atrophy would be diminished [13]. Moreover, the sural nerve will be protected by the gastrocnemius muscle during endoscopic recession of the muscle-bound portion of the gastrocnemius aponeurosis and the risk of iatrogenic sural nerve injury will be lessened [13, 24].

Clinical relevance of this report is that it suggests that resection of muscle bound portion rather than the muscle void portion of the gastrocnemius aponeurosis is a more appropriate approach of endoscopic gastrocnemius aponeurosis recession.

## CONCLUSION

There are great anatomical variations of the gastrocnemius insertion. Resection of muscle bound portion of the gastrocnemius aponeurosis is a more appropriate approach of endoscopic gastrocnemius aponeurosis recession.

## Figures and Tables

**Table 1:** Summary of the distance from the junction point to the posterior calcaneal tubercle and the distance between the junction point and the lowest point of the gastrocnemius muscle.

**Specimen no.**	**Laterality**	**Distance between the Tibial plateau and the calcaneal tubercle (mm): A**	**Distance between the junction point and the calcaneal tubercle (mm): B**	**B/A**	**The distance of the junction point distal to the lowest border of gastrocnemius muscle (mm)**
1	L	390	180	0.46	0
2	L	380	175	0.46	5
3	R	370	175	0.47	-5
4	L	380	190	0.5	10
5	R	390	120	0.31	25
6	L	380	145	0.38	0
7	L	415	155	0.37	5
8	R	340	185	0.54	-5
9	R	410	170	0.41	-10
10	R	350	175	0.5	0
11	L	340	185	0.54	-25
12	R	335	160	0.48	0
